# Impact of postoperative radiotherapy and chemoradiotherapy on survival outcomes in oral squamous cell carcinoma patients with pN1 neck disease

**DOI:** 10.1007/s00784-025-06730-6

**Published:** 2026-01-10

**Authors:** Ann-Kristin Struckmeier, Ralf Smeets, Cordula Petersen, Christian Betz, Waldemar Wilczak, Martin Gosau

**Affiliations:** 1 Department of Oral and Maxillofacial Surgery, University Medical Center Hamburg- Eppendorf, Martinistraße 52, Hamburg, 20246 Germany; 2 Department of Oral and Maxillofacial Surgery, Division of Regenerative Orofacial Medicine, University Medical Center Hamburg-Eppendorf, Hamburg, Germany; 3 Department of Radiotherapy and Radiation Oncology, University Medical Center Hamburg-Eppendorf, Hamburg, Germany; 4 Department of Otorhinolaryngology, University Medical Center Hamburg- Eppendorf, Hamburg, Germany; 5 Department of Pathology, University Medical Center Hamburg-Eppendorf, Hamburg, Germany

**Keywords:** Oral squamous cell carcinoma, Head and neck squamous cell carcinoma, Neck dissection, Lymph node metastases, Radiotherapy, Chemoradiotherapy

## Abstract

**Background:**

The lymph node management of oral squamous cell carcinoma (OSCC) patients with pN1 neck remains a clinical challenge. This study investigates the impact of different treatment modalities – surgery alone, surgery with postoperative radiotherapy (PORT), and surgery with postoperative chemoradiotherapy (PCRT) – on recurrence and survival outcomes in OSCC patients with pN1 neck.

**Methods:**

A retrospective cohort study was conducted on 98 patients who underwent tumor resection and neck dissection, with a subset receiving adjuvant therapy, between 2011 and 2020. Clinicopathological characteristics were examined for associations with treatment modality using chi-square test. Kaplan-Meier survival curves for overall survival (OS) and recurrence-free survival (RFS), along with the log-rank test, were employed to assess survival over a 10-year period. The prognostic significance of clinicopathological factors and treatment modalities was evaluated using Cox proportional hazards models.

**Results:**

The addition of PORT following surgery significantly reduced the risk of recurrence (HR = 0.280, *p* = 0.010) and enhanced RFS, particularly at 10 years (*p* = 0.010). PORT was also associated with a statistically significant improvement in 5-year-OS (*p* = 0.031) compared to surgery alone, with a trend toward improved OS at 10 years (*p* = 0.175). In contrast, surgery alone did not yield comparable 10-year survival outcomes. Additionally, the inclusion of PCRT did not show a distinct survival advantage over the combination of surgery and PORT.

**Conclusion:**

Our results indicate that PORT may be considered the standard adjuvant treatment for these patients, as it improves RFS, particularly in the long term, while the addition of chemotherapy does not provide significant additional benefits over PORT alone.

## Introduction

Oral squamous cell carcinoma (OSCC) is one of the most common malignancies worldwide, accounting for a significant proportion of head and neck cancers [1]. Approximately 30%−40% of patients with OSCC develop lymph node metastases, with the presence of cervical metastases significantly correlating with a substantial reduction in survival rates [2, 3].

Currently, the surgical management of OSCC typically involves radical resection of the primary tumor in combination with appropriate neck dissection, which is regarded as the standard treatment approach [2]. The decision to administer postoperative radiotherapy (PORT) remains a topic of ongoing debate, particularly in cases of pN1 neck, defined by a single ipsilateral lymph node metastasis without extranodal extension (ENE), as outlined in the 8th UICC classification [4]. Although the current guidelines recommend adjuvant radiotherapy for patients with higher-risk features, such as multiple nodal metastases or ENE, the benefits of PORT in low-risk cases, such as patients with pN1 neck, are not clearly defined [5–8]. However, some studies suggest that PORT could potentially improve locoregional control and survival in pN1 neck cases, while others report no clear survival advantage [9, 10]. Nonetheless, the impact of PORT on the quality of life in these patients remains a critical consideration, as the potential side effects and long-term consequences of treatment may outweigh the benefits of improved survival outcomes [11–14].

The role of chemotherapy, particularly in combination with radiotherapy, is another important factor in the management of OSCC, though its role in patients with pN1 neck remains less clear. Chemotherapy is often employed as part of postoperative chemoradiotherapy (PCRT) for more advanced or high-risk cases, such as those with ENE or multiple nodal metastases [15–19]. While chemotherapy in combination with radiotherapy has demonstrated survival benefits in higher-risk OSCC patients [16], its potential role in patients with pN1 neck without adverse features remains uncertain.

This study aims to evaluate the role of different treatment regimes in patients with OSCC and a single ipsilateral cervical lymph node metastasis without ENE (pN1) regarding survival outcomes and recurrence. By comparing patients treated with surgery alone versus those receiving PORT or PCRT, we aim to provide evidence to guide clinical decisions in this patient cohort.

## Materials and methods

### Study design and participants

This retrospective study included patients diagnosed with primary OSCC and pN1 neck (solitary, ipsilateral neck node metastasis) who underwent radical tumor resection and neck dissection at a high-volume tertiary center in Germany between January 1, 2011, and December 31, 2020. Both the last date of follow-up and the time point of data analysis were July 29, 2024. The treatment protocol adhered to the current German guidelines, and all procedures were performed following recommendations from multidisciplinary oncology board meetings.

Neck dissection was conducted according to a standardized protocol. For patients without clinically detectable neck metastases, a unilateral selective neck dissection (SND) involving levels I to IV was performed. In cases with tumors located at or near the midline, a bilateral SND was carried out. If ipsilateral lymph node metastases were identified preoperatively, intraoperatively (via frozen section), or postoperatively, a modified radical neck dissection (MRND) was performed on the affected side, along with a contralateral SND. In patients with contralateral lymph node metastases, bilateral MRND was performed. The decision regarding adjuvant therapy was based on individual patient risk factors and followed the German treatment guidelines, which recommend adjuvant treatment for patients with close or positive surgical margins, extranodal extension, multiple lymph node metastases, perineural or lymphatic invasion, advanced T stage, or other adverse histopathological features.

Patients received platin-based chemotherapy.

The exclusion criteria included patients with recurrent OSCC, squamous cell carcinoma of the lip, those who had received neoadjuvant immunotherapy, patients who declined neck dissection, individuals who underwent sentinel lymph node biopsy, and those who had limited neck dissection due to severe comorbidities.

The primary endpoint was recurrence-free survival (RFS), as regional control represents the most clinically relevant outcome in pN1 OSCC, while overall survival (OS) was defined as the secondary endpoint.

The study protocol was approved by the Ethics Committee of the Ärztekammer Hamburg (ethics vote: 2024–101379-BO-ff), and written informed consent was not required per national and institutional regulations due to the retrospective nature of the study. The manuscript adhered to STROBE guidelines.

### Clinicopathological characteristics

Clinicopathological data were extracted from patient medical records, including age, sex, tumor localization, TNM classification, depth of invasion, ENE, grading, resection margins, and the presence of perineural, vascular, and lymphatic invasion. We also recorded the dates of surgery, last follow-up, recurrence, and death.

During the study period, the TNM classification was updated from the 7th to the 8th edition. Patients initially classified according to the 7th edition were reclassified according to the 8th edition (2018) to ensure consistency [4]. As ENE was not considered in the 7th TNM classification, some patients classified as pN1 under this system had to be excluded due to the presence of ENE (*n* = 11).

#### Statistical analysis

Data analysis was conducted using SPSS software, version 28.0 (SPSS, IBM, Chicago, IL, USA). Correlation analysis was performed utilizing the chi-square test. Recurrence-free survival (RFS) and overall survival (OS) were estimated using the Kaplan-Meier method, and comparisons of survival outcomes between patients undergoing different treatment modalities were analyzed using the log-rank test. RFS was defined as the interval from surgery to the occurrence of local or regional recurrence, with data censored at the last follow-up in cases without recurrence. OS was defined as the time from surgical resection to death from any cause, with data censored at the last follow-up for patients who were still alive. For identifying prognostic factors of survival, univariate Cox analysis was performed, followed by a multivariate Cox analysis that incorporated factors showing significance in the univariate analysis.

Figures were generated using SPSS.

A p-value of < 0.05 was considered statistically significant.

## Results

### Clinicopathological characteristics of the cohort

The study included 98 patients, with a slightly higher proportion of males (57; 58.2%) than females (41; 41.8%). The age distribution was roughly balanced between patients < 65 and ≥ 65 years, with 53 patients (54.1%) under 65 years and 45 (45.9%) aged 65 years or older.

Pathological staging revealed that T2 was the most common T stage, observed in 37 patients (37.8%), followed by T4a in 28 patients (28.6%), T1 in 21 patients (21.4%), and T3 in 12 patients (12.2%). Clinically, T2 was also the most frequent stage, present in 37 cases (37.8%). Other clinical T stages included T1 in 12 cases (12.2%), T3 in 7 cases (7.1%), T4a in 22 cases (22.5%), and T4b in a single case (1.0%).

Regarding clinical N stage, nearly half of the patients (46; 47.0%) were node-negative (N0). Among the remaining patients, N1 was observed in 20 cases (20.4%), N2b in 9 cases (9.2%), N2c in 4 cases (4.1%), and N2a and N3b in 1 case each (1.0%). All patients in the cohort were classified as M0, indicating the absence of distant metastases at the time of diagnosis.

Tumor grading showed that the majority of cases (67; 68.4%) were moderately differentiated (G2), while 23 (23.5%) were poorly differentiated (G3), and 3 (3.1%) were well-differentiated (G1). Lymphatic invasion was identified in 37 patients (37.8%), while 61 patients (62.2%) showed no evidence of such invasion. Vascular invasion was rare, occurring in only 3 cases (3.1%). Perineural invasion was present in 20 patients (20.4%), whereas 78 patients (79.6%) had no signs of perineural involvement. Resection margins were negative (R0) in 96 cases (98.0%), with only 2 patients (2.0%) having positive margins (R1).

The clinical outcomes demonstrated a recurrence rate of 26.5%, with 26 patients experiencing recurrence, while 72 patients (73.5%) showed no signs of recurrence during follow-up. The recurrences were distributed as follows: 11 patients (11.2%) showed a local recurrence, 6 patients (6.1%) a regional recurrence, and 9 patients (9.2%) a locoregional recurrence. Distant metastases during follow-up were rare, occurring in only 5 patients (5.1%).

Regarding treatment, 35 patients (35.7%) underwent surgery alone, 19 patients (19.4%) received surgery with PCRT, and 44 patients (44.9%) underwent surgery followed by PORT. In the surgery-only group, 7 patients (20.0%) declined adjuvant therapy due to advanced age or multiple comorbidities that rendered adjuvant treatment unsafe.

The median follow-up time was 65 months (range, 8–122 months), and the mean follow-up time was 68.4 ± 27.1 months.

Further details on the clinicopathological characteristics of the investigated cohort are provided in Table 1..

**Table 1.** Clinicopathological characteristics of the cohort

Variable	Category	*n* (%)
Sex	**Female**	41 (41.8)
	**Male**	57 (58.2)
Age at surgery	**< 65 years**	53 (54.1)
	**≥ 65 years**	45 (45.9)
Pathological T stage	**T1**	21 (21.4)
	**T2**	37 (37.8)
	**T3**	12 (12.2)
	**T4a**	28 (28.6)
Clinical T stage	**T1**	12 (12.2)
	**T2**	37 (37.8)
	**T3**	7 (7.1)
	**T4a**	22 (22.5)
	**T4b**	1 (1.0)
	**Missing**	17 (17.4)
Clinical N stage	**N0**	46 (47.0)
	**N1**	20 (20.4)
	**N2a**	1 (1.0)
	**N2b**	9 (9.2)
	**N2c**	4 (4.1)
	**N3b**	1 (1.0)
	**Missing**	17 (17.4)
M stage	**M0**	98 (100.0)
Grading	**G1**	3 (3.1)
	**G2**	67 (68.4)
	**G3**	23 (23.5)
	**Gx**	5 (5.1)
Lymphatic invasion	**L0**	61 (62.2)
	**L1**	37 (37.8)
Vascular invasion	**V0**	95 (96.9)
	**V1**	3 (3.1)
Perineural invasion	**Pn0**	78 (79.6)
	**Pn1**	20 (20.4)
Resection margin	**R0**	96 (99.0)
	**R1**	2 (2.0)
Depth of invasion	**≤ 5 mm**	24 (24.50)
	**6–10 mm**	36 (36.73)
	**≥ 11 mm**	38 (38.77)
Recurrence	**No**	72 (73.5)
	**Yes**	26 (26.5)
Occurrence of distant metastases during observation period	**No**	93 (94.9)
	**Yes**	5 (5.1)
Localization	**Tongue**	28 (28.6)
	**Floor of the mouth**	28 (28.6)
	**Lower jaw**	19 (19.4)
	**Upper jaw**	4 (4.1)
	**Hard palate**	10 (10.2)
	**Buccal mucosa**	9 (9.2)
Therapy	**Surgery only**	35 (35.7)
	**Surgery + PCRT**	19 (19.4)
	**Surgery + PORT**	44 (44.9)

Abbreviations: *T stage* tumor stage, *M* metastasis, *PCRT *postoperative chemoradiotherapy, *PORT* postoperative radiotherapy.

*Gx* means that grading was not reported

### Correlation between clinicopathological characteristics and pN1 neck

 Chi-square test was performed to assess the associations between clinicopathological characteristics and therapy types. In terms of sex, females and males were evenly distributed across therapy types, with no significant association observed (p = 0.745). Age at surgery was significantly associated with therapy type (p = 0.046). Patients under 65 years of age were more likely to receive surgery only (34.0%) or surgery with PCRT (28.3%), while those aged 65 years or older predominantly received PORT (53.3%).

 Pathological T stage showed a significant association with therapy type (p = 0.049). Patients with T1 tumors most frequently underwent surgery only (61.9%), while those with advanced stages, such as T3 and T4a, were more likely to receive PORT (58.3% and 57.1%, respectively).

 Grading did not show a significant relationship with therapy type (p = 0.342), although poorly differentiated tumors (G3) tended toward PORT (65.2%). Other histopathological features, including lymphatic invasion (p= 0.477), vascular invasion (p = 0.816), and perineural invasion (p = 0.188), did not show significant associations with therapy type. However, patients with perineural invasion were slightly more likely to receive PORT (50.0%). Similarly, resection margins did not demonstrate a significant association (p = 0.376), though patients with positive margins tended toward PORT (50.0%).

 Recurrence was significantly associated with therapy type (p = 0.033). Among patients without recurrence, a higher proportion had received PORT (52.8%), whereas those with recurrence were more likely to have undergone surgery only (50.0%). The presence of distant metastases during follow-up did not show a significant association with therapy type (p = 0.385). Regarding tumor localization, no significant association was found with therapy type (p = 0.879). Tumors located in the floor of the mouth or lower jaw were evenly distributed across all therapy types, while those in the hard palate showed a slight preference for surgery only (50.0%) and PORT (30.0%). Please see Table 2 for further details.

**Table 2:** Association between clinicopathological variables and type of therapy

Variable	Characteristic	Type of therapy			
		Surgery only n (%)	Surgery + PCRT n (%)	Surgery + PORT n (%)	p value
Sex	**Female**	13 (31.7)	9 (22.0)	19 (46.3)	0.745
	**Male**	22 (38.6)	10 (17.5)	25 (43.9)	
Age at surgery	**< 65 years**	18 (34.0)	15 (28.3)	20 (37.8)	0.046*
	**≥ 65 years**	17 (37.8)	4 (9.0)	24 (53.3)	
Pathological T stage	**T1**	13 (61.9)	3 (14.3)	5 (23.8)	0.049*
	**T2**	15 (40.5)	6 (16.2)	16 (43.2)	
	**T3**	2 (16.7)	3 (25.0)	7 (58.3)	
	**T4a**	5 (17.9)	7 (25.0)	16 (57.1)	
Grading	**G1**	1 (33.3)	1 (33.3)	1 (33.3)	0.342
	**G2**	26 (38.8)	13 (19.4)	28 (41.8)	
	**G3**	6 (26.1)	2 (8.7)	15 (65.2)	
Lymphatic invasion	**L0**	21 (34.4)	10 (16.4)	30 (49.2)	0.477
	**L1**	14 (37.8)	9 (24.3)	14 (37.8)	
Vascular invasion	**V0**	34 (35.8)	18 (19.0)	43 (45.3)	0.816
	**V1**	1 (33.3)	1 (33.3)	1 (33.3)	
Perineural invasion	**Pn0**	31 (39.7)	13 (16.7)	34 (43.6)	0.188
	**Pn1**	4 (20.0)	6 (30.0)	10 (50.0)	
Resection margin	**R0**	29 (34.1)	14 (16.5)	42 (49.4)	0.376
	**R1**	0 (0.0)	1 (50.0)	1 (50.0)	
Recurrence	**No**	22 (30.6)	12 (16.7)	38 (52.8)	0.033*
	**Yes**	13 (50.0)	7 (26.9)	6 (23.1)	
Occurrence of distant metastases during observation period	**No**	33 (35.5)	17 (16.3)	43 (46.2)	0.385
	**Yes**	2 (40.0)	2 (40.0)	1 (20.0)	
Localization	**Tongue**	13 (46.4)	4 (14.3)	11 (39.3)	0.816
	**Floor of the mouth**	8 (28.6)	7 (25.0)	13 (46.43)	
	**Lower jaw**	6 (31.6)	4 (21.1)	9 (43.4)	
	**Upper jaw**	1 (25.0)	0 (0.0)	3 (75.0)	
	**Hard palate**	5 (50.0)	2 (20.0)	3 (30.0)	
	**Buccal mucosa**	2 (22.2)	2 (22.2)	5 (55.6)	

A p value < 0.05 was considered statistically significant. Statistically significant differences are marked with an asterisk

*Abbreviations*: *PCRT* postoperative chemoradiotherapy, *PORT* postoperative radiotherapy

###  Univariate and multivariate analysis for recurrence-free survival

 The univariate analysis demonstrated that the type of therapy, vascular invasion, and resection margin were significantly associated with RFS (p < 0.05).

 Patients treated with surgery followed by PORT had a significantly lower risk of recurrence compared to those treated with surgery alone (HR = 0.280, 95% CI: 0.106-0.737, p = 0.010). Similarly, the presence of vascular invasion was associated with a markedly increased risk of recurrence (HR = 13.004, 95% CI: 2.727-62.002, p = 0.001). Positive resection margins also showed a strong association with recurrence, with a HR of 24.987 (95% CI: 4.175-149.567, p < 0.001). Other factors, including age, sex, pathological T category, tumor grading, lymphatic invasion, and perineural invasion, did not reach statistical significance in the univariate analysis (p > 0.05).

 In the multivariate analysis, PORT remained independently associated with improved RFS (HR = 0.279, 95% CI: 0.098-0.789, p = 0.016), confirming its protective effect. Although vascular invasion and resection margins were significant in univariate analysis, their association with RFS was attenuated in the multivariate model, with p-values of 0.251 and 0.072, respectively. Please refer to Table 3.

**Table 3:** Univariate and multivariate analysis of recurrence-free survival

Clinicopathological characteristics	Univariate analysis			Multivariate analysis				
	HR	95% CI	*p* value	HR	95% CI		*p* value	
Age < 65 vs. ≥ 65 years	0.984	0.435–2.066	0.894					
Sex	2.189	0.993–4.827	0.052					
pT category								
T1	1							
T2	2.202	0.621–7.810	0.222					
T3	2.146	0.429–10.743	0.353					
T4a	1.848	0.490–6.969	0.364					
Type of therapy								
Surgery only	1			1				
Surgery + PCRT	0.793	0.316–1.989	0.621	0.318		0.076–1.327		0.116
Surgery + PORT	0.280	0.106–0.737	0.010*	0.279		0.098–0.789		0.016*
Grading	0.501	0.172–1.461	0.206					
Lymphatic invasion	1.458	0.630–3.376	0.379					
Vascular invasion	13.004	2.727–62.002	0.001*	4.822	0.328–70.822		0.251	
Perineural invasion	1.189	0.428–3.304	0.739					
Resection margin	24.987	4.175–149.567.175.567	< 0.001*	13.514		0.792–230.700		0.072

A p value < 0.05 was considered statistically significant. Statistically significant differences are marked with an asterisk

*Abbreviations*: *CI* confidence interval, *HR* hazard ratio, *PCRT* postoperative chemoradiotherapy, *PORT* postoperative radiotherapy, *RFS* recurrence-free survival

###  Univariate and multivariate analysis for overall survival

 The univariate analysis revealed that type of therapy and resection margins were significantly associated with OS (p < 0.05). Patients treated with surgery followed by PORT exhibited a trend toward improved survival compared to those treated with surgery alone (HR = 0.504, 95% CI: 0.240-1.058, p = 0.050). Additionally, positive resection margins were associated with a markedly worse OS (HR = 9.386, 95% CI: 2.042-43.136, p = 0.004). Other clinicopathological factors, including age, sex, pathological T category, tumor grading, lymphatic invasion, vascular invasion, and perineural invasion, were not significantly associated with OS in the univariate analysis (p > 0.05).

 In the multivariate analysis, the protective effect of PORT on OS became more pronounced and reached statistical significance (HR = 0.361, 95% CI: 0.165-0.790, p = 0.011), confirming its independent role in improving survival outcomes. Furthermore, positive resection margins remained a significant predictor of worse survival in the adjusted model (HR = 14.036, 95% CI: 2.886-68.258, p = 0.001). Please refer to Table 4.

**Table 4:** Univariate and multivariate analysis of overall survival

Clinicopathological characteristics	Univariate analysis			Multivariate analysis				
	HR	95% CI	*p* value	HR	95% CI		*p* value	
Age < 65 vs. ≥ 65 years	1.572	0.823–3.003	0.171					
Sex	1.202	0.629–2.297	0.577					
pT category								
T1	1							
T2	0.935	0.403–2.171	0.876					
T3	1.045	0.317–3.450	0.942					
T4a	0.639	0.253–1.612	0.343					
Type of therapy								
Surgery only	1			1				
Surgery + PCRT	0.759	0.327–1.763	0.521	0.423		0.158–1.132		0.087
Surgery + PORT	0.504	0.240–1.058	0.050	0.361		0.165–0.790		0.011*
Grading	1.150	0.543–2.437	0.715					
Lymphatic invasion	1.063	0.536–2.109	0.861					
Vascular invasion	5.542	0.680–45.136.680.136	0.110					
Perineural invasion	1.1355	0.623–2.945	0.443					
Resection margin	9.386	2.042–43.136	0.004*	14.036		2.886–68.258		0.001*

A p value < 0.05 was considered statistically significant. Statistically significant differences are marked with an asterisk

*Abbreviations:* *HR * hazard ratio, *CI* confidence interval, *PCRT* postoperative chemoradiotherapy, *PORT* postoperative radiotherapy, *OS* overall survival

###  Recurrence-free survival

 The 5-year-RFS was highest among patients who underwent surgery followed by PORT. However, this outcome only demonstrated a trend toward improved survival in the surgery plus PORT group, without reaching statistical significance when compared to the surgery-alone group (p= 0.092) or the surgery combined with PCRT group (p = 0.096). Additionally, no significant difference in RFS at 5 years was found between the surgery-alone group and the surgery with PCRT group (p > 0.05).

**Fig. 1:**
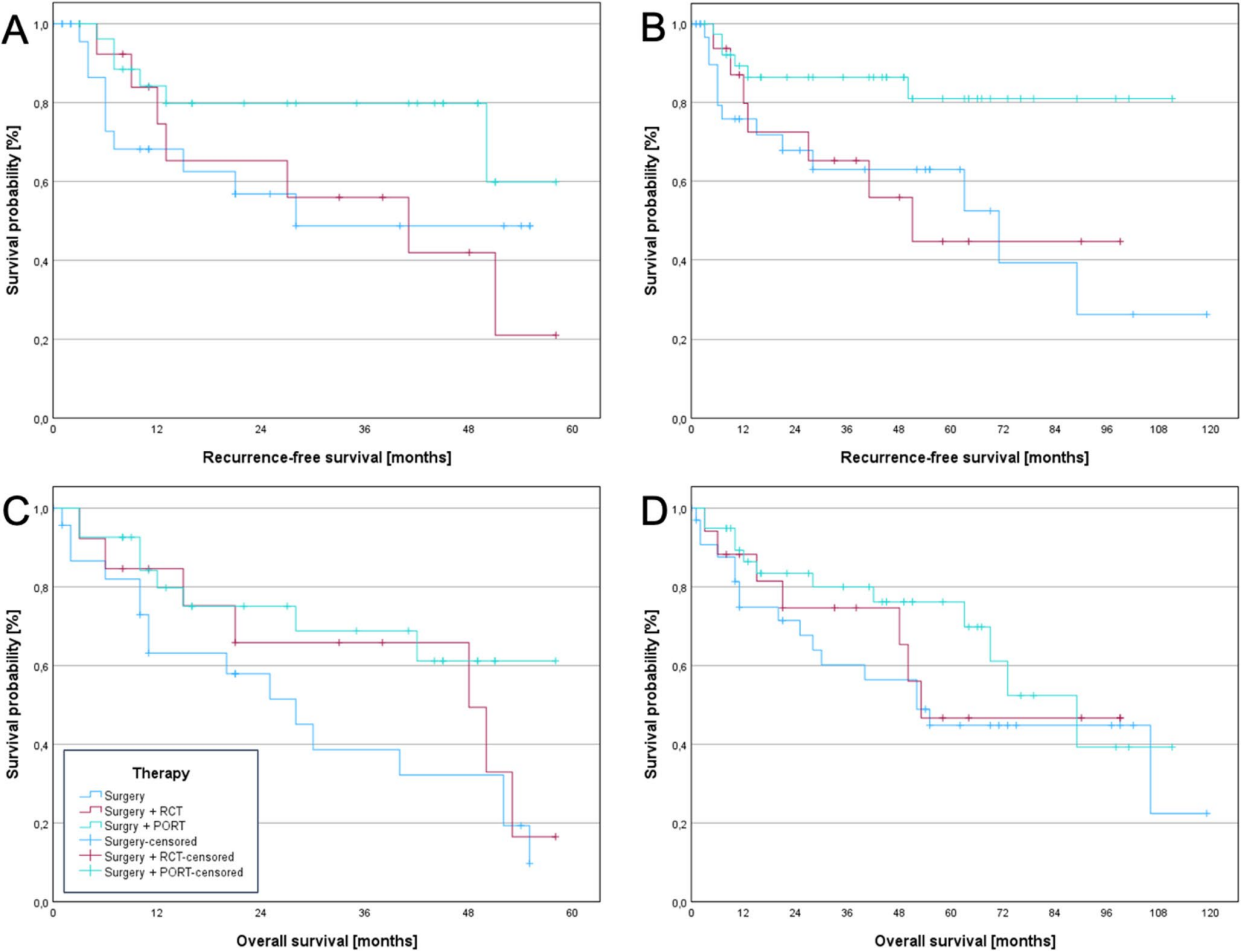
Comparison of 5- and 10-year-recurrence-free survival and overall survival among different treatment modalities in oral squamous cell carcinoma patients with pN1 neck. (**A**) 5-Year-Recurrence-free survival: Group 3 (surgery + PORT) exhibited the highest RFS, although no statistically significant differences were found when compared to group 1 (surgery only, *p* = 0.092) or group 2 (surgery + RCT, *p* = 0.096). No significant difference in RFS was observed between group 1 and group 2 (*p* > 0.05) (**B**) 10-Year-Recurrence-free survival: A significant improvement in RFS was observed for group 3 (surgery + PORT) compared to both group 1 (surgery only, *p* = 0.010) and group 2 (surgery + RCT, *p* = 0.030). No significant difference was found between group 1 and group 2 (*p* = 0.855) (**C**) 5-Year-Overall survival: A significant difference in overall survival was found between group 3 (surgery + PORT) and group 1 (surgery only, *p* = 0.038). No significant differences were observed between group 1 (surgery only) and group 2 (surgery + RCT, *p* = 0.412), or between group 3 (surgery + PORT) and group 2 (*p* = 0.426) (**D**) 10-Year-Overall survival: No significant differences were found between the groups (*p* > 0.05). However, a trend toward slightly better survival was noted in group 3 (surgery + PORT) compared to group 1 (surgery only), with a p-value of 0.175

 At 10 years, a significant improvement in RFS was observed in the surgery + PORT group compared to both the surgery-alone group (p = 0.010) and the surgery + PCRT group (p = 0.030). However, no significant difference in RFS was found between the surgery-alone group and the surgery + PCRT group (p = 0.855). Please refer to Figure1A and 1B for additional information.

###  Overall survival

 Regarding 5-year-OS, a significant difference was observed between the surgery with PORT group and the surgery-alone group (p = 0.038). No significant differences in OS were found between the surgery-alone group and the surgery with PCRT group (p = 0.412), or between the surgery with PORT group and the surgery with PCRT group (p = 0.426).

 At 10 years, no significant differences in OS were observed between the treatment groups (p > 0.05). However, a trend toward slightly better survival was noted in the surgery with PORT group compared to the surgery-alone group, with a p-value of 0.175. Please refer to Figure 1C and 1D for additional information.

## Discussion

The role of PORT in the management of OSCC patients with pN1 neck, particularly in the absence of other adverse pathological features, remains a subject of ongoing debate. Additionally, the potential benefits of PCRT in improving survival outcomes are also under investigation. This study examines the efficacy of different treatment strategies – surgery alone, surgery with PORT, and surgery with PCRT – for managing the pN1 neck in OSCC patients. The results highlight significant differences in the effects of these treatment modalities on recurrence and survival, providing valuable insights for clinical decision-making in this patient population.

Initially, we assessed the factors influencing treatment selection. Age at surgery and pathological T stage emerged as significant predictors of treatment modality. Notably, patients under 65 years of age were more likely to undergo surgery alone or surgery with PCRT, while those aged 65 or older were predominantly treated with PORT, especially in cases with advanced tumor stages such as T3 or T4a (*p* < 0.05). Additionally, recurrence was strongly associated with treatment choice. Patients who did not experience recurrence were more likely to have received PORT, whereas those who experienced recurrence were more likely to have been treated with surgery alone (*p* < 0.05).

In terms of RFS, the addition of PORT to surgery demonstrated the most favorable long-term outcomes, particularly at 10 years. At this time point, patients who underwent surgery with PORT had significantly better RFS compared to those treated with surgery alone (*p* = 0.010) or surgery with PCRT (*p* = 0.030). These findings suggest that PORT provides a substantial long-term benefit in preventing recurrence. In contrast, at 5 years, no significant differences in RFS were observed between the treatment groups (*p* > 0.05).

Furthermore, no significant difference in 5-Year-RFS was found between the surgery-only and surgery with PCRT groups (*p* > 0.05), suggesting that the addition of chemotherapy to radiotherapy does not provide a clear advantage over surgery alone in the medium term.

Univariate analysis confirmed that PORT significantly reduced the risk of recurrence compared to surgery alone (HR = 0.280, *p* = 0.010). Multivariate analysis further supported this finding, with PORT remaining independently associated with improved RFS (HR = 0.279, *p* = 0.016). Additionally, while factors such as vascular invasion and positive resection margins were significant in the univariate analysis, their impact on RFS was diminished in the multivariate model, highlighting the moderating role of PORT in improving outcomes.

Consistent with our findings, most studies on pN1 neck in OSCC patients suggest that PORT reduces the risk of regional recurrence. Barry et al. performed a matched-pair analysis on 90 OSCC patients, including 30 with pN1, comparing those who received surgery with PORT to those treated with surgery alone. Their study demonstrated a significant improvement in locoregional tumor control in the PORT group [13].

Similarly, Liu et al. evaluated the treatment outcomes in a cohort of 432 OSCC patients, 216 of whom received surgery alone and 216 received surgery with PORT. They observed a significantly improved RFS in patients who underwent surgery with PORT, with a higher 5-year RFS rate in this group compared to the surgery-only group [20].

Kämmerer et al., in a multicenter trial involving 209 patients, found that PORT had a positive impact on progression-free survival, delaying disease progression relative to non-RT treatment. However, they also noted that patients who received PORT reported a significantly reduced quality of life up to three years post-treatment compared to the observation group [21]. In contrast to previous findings and our own results, Tsai et al. found no significant differences in neck recurrence rates or RFS between patients who underwent surgery alone and those treated with surgery and PORT (7.7% vs. 15.8%, *p* > 0.05) [9].

Regarding OS, patients who received surgery followed by PORT demonstrated significantly better survival at 5 years compared to those who underwent surgery alone (*p* = 0.038). However, no significant differences in OS were observed between the surgery-only and surgery with PCRT groups (*p* = 0.412) or between the surgery with PORT and surgery with PCRT groups (*p* = 0.426). These results suggest that PORT provides a survival advantage over surgery alone, but the addition of chemotherapy does not significantly enhance survival at this early time point.

At 10 years, while no statistically significant differences in OS were observed (*p* > 0.05), a trend toward improved survival in the surgery + PORT group compared to surgery alone persisted, though it did not reach statistical significance (*p* = 0.175).

Univariate analysis showed that PORT was associated with a trend toward improved survival compared to surgery alone (HR = 0.504, *p* = 0.050). In multivariate analysis, PORT significantly improved OS (HR = 0.361, *p* = 0.011), confirming its beneficial impact on survival outcomes. However, positive resection margins emerged as a strong predictor of poor survival (HR = 14.036, *p* = 0.001), underscoring the importance of achieving negative margins to improve prognosis. In contrast, the observed survival benefit of PORT was independent of T stage (*p* > 0.05).

The addition of PCRT did not demonstrate a clear survival benefit in the multivariate analysis (HR = 0.423, *p* = 0.087), suggesting that while PCRT may offer some advantages, it does not provide significant improvements in OS over PORT alone. However, it is important to note, that in our cohort patients who received PCRT tended to present with more adverse histopathological features, such as close or narrow margins, perineural invasion, or higher T stage. These unfavorable characteristics likely contributed to the lack of observed benefit from concurrent chemotherapy in this subgroup.

In contrast to our findings, several studies have failed to demonstrate a significant difference in OS. For instance, Liu et al. found no substantial difference in OS between patients who underwent surgery alone and those treated with surgery plus PORT, with similar 5-year OS rates in both groups [20]. Similarly, in a multicenter trial involving 209 patients, Kämmerer et al. reported that PORT did not significantly affect OS in this cohort [21]. On the contrary, Barry et al. observed that the addition of PORT suggested a survival benefit; however, this finding did not reach statistical significance [13]. These inconsistencies may stem from variations in patient populations and treatment protocols. For example, Chen et al. analyzed a cohort of 1,467 OSCC patients and found that PORT was associated with improved OS, with a hazard ratio of 0.76 (95% CI, 0.63–0.92). in addition, a survival benefit was observed in patients younger than 70 years, and those with pT2 disease also benefitted from PORT. In contrast, no significant survival advantage was noted for patients aged 70 years or older [22]. Moreover, Shrime et al. demonstrated that PORT conferred survival benefits only in patients with T2 tongue and tumors located at the floor of the mouth, with no observed benefit in other subsites [23]. Similarly, Tsai et al. as well as Li et al. [24] reported no significant differences in overall or disease-specific survival and suggested that, in the absence of additional adverse pathological features, omission of adjuvant therapy did not compromise patient outcomes [25]. On the contrary, an analysis by Suresh and Crameri, which included 1,909 patients (898 of whom received PORT), indicated a survival benefit in the overall cohort, with an adjusted hazard ratio of 0.82 (95% CI 0.72–0.94). This benefit was evident regardless of the adequacy of neck dissection, and patients with lymph node metastases larger than 10 mm experienced greater benefit from PORT compared to those with smaller metastases [26].

The EHNS-ESMO-ESTRO guideline suggests that patients with pN1 disease and no other risk factor do not require PORT if at least 15 nodes have been analysed [8]. Evans et al. currently recommend administering PORT when the metastatic lymph node diameter exceeds 10 mm and the lymph node yield is below 18 [27].

While the potential benefits of PORT in reducing regional recurrence are well-documented, it is essential to consider the associated implications for patients’ quality of life. Several studies, including those by Bekiroglu et al. and Barry et al., have highlighted the significant short-term and long-term functional impairments that patients may experience following PORT. These impairments include pain, dysphagia, speech disturbances, xerostomia, and restricted mouth opening, all of which can substantially affect patients’ daily functioning and overall well-being [12, 13, 20].

The benefit of adding chemotherapy to radiotherapy remains a topic of debate. In contrast to our findings, a cohort study by Lee et al., which included 1,598 patients with pT1/2 N1 HNSCC without positive margins or ENE, demonstrated that patients receiving PCRT had a 13.8% improvement in survival compared to those treated with PORT. However, no significant difference in survival was observed between patients treated with PORT and those treated with surgery alone [28]. Similarly, Chang et al. reported that 5-year-OS rates for patients treated with surgery alone, PORT, and PCRT were 62.2%, 58.7%, and 71.1%, respectively (*P* = 0.03). PCRT was associated with significantly improved survival compared to PORT in the overall group (*p* = 0.008). However, this benefit was particularly evident in the pT2 group, where PCRT resulted in significantly better survival rates than PORT (*p* = 0.001). In contrast, no significant survival differences were observed between the two treatment approaches for patients with pT1 disease [29].

###  Limitations of this study

While this study provides valuable insights into the management of the pN1 neck in patients with OSCC, several limitations should be acknowledged. First, the retrospective design and the relatively small sample size may limit the generalizability of the findings. Although only few studies with larger case numbers are available in this specific setting, the results of our subgroup analyses in particular should be interpreted with caution. For instance, the small number of patients with positive resection margins resulted in wide confidence intervals for the corresponding hazard ratios.

Furthermore, certain patient-related factors, such as performance status and comorbidities, could not be systematically assessed and may have influenced both treatment decisions and outcomes. Although all patients were treated according to the current German guidelines and surgical margin assessment (“clear” vs. “close” vs. “positive”) was performed in close interdisciplinary agreement between surgeons and pathologists, this detailed differentiation could not be reliably reconstructed retrospectively in all cases. Consequently, only the R classification (R0/R1) was consistently available for statistical analysis, which represents an additional limitation of this study.

Moreover, due to the retrospective study design, quality-of-life outcomes could not be evaluated. Therefore, further studies with larger cohorts, preferably with prospective designs, are needed to confirm these findings and to better define the role of PORT and PCRT in improving long-term survival in this patient population Nevertheless, the strengths of this study include the uniform classification of all patients according to the 8th UICC staging system, the thorough pathological characterization of the cohort, and the long follow-up period of up to 10 years, providing valuable insights into the long-term prognostic impact of adjuvant treatment in patients with pN1 OSCC.

## Conclusion

In the management of OSCC patients with pN1 neck, PORT is associated with improved RFS and OS, especially in the long term. Surgery combined with PORT provides the most significant survival benefit, while the addition of PCRT does not show a clear advantage over surgery alone or surgery with PORT. Our results indicate that PORT may be considered the preferred adjuvant treatment for OSCC patients with pN1 neck disease, while PCRT may be reserved for those with specific high-risk characteristics. Nonetheless, impairments in quality of life should be taken into account.

## Data Availability

The data that support the findings of this study are available from the corresponding author upon reasonable request.

## Abbreviations

ASCO: American Society of Clinical Oncology
CI: confidence interval
ENE: extranodal extension
HR: hazard ratio
MRND: modified radical neck dissection
OS: overall survival
OSCC: oral squamous cell carcinoma
PCRT: postoperative chemoradiotherapy
PORT: postoperative radiotherapy
RFS: recurrence-free survival
SND: selective neck dissection
SLNB: sentinel lymph node biopsy
UICC: Union for International Cancer Control
